# 

**Published:** 1907-12-21

**Authors:** 


### BOOKS RECEIVED.

The Office of "Modern Astrology."

"Medical Astrology." By Heinrich Däath.

Baillière, Tindall, and Cox.

"Cancer." By G. Sherman Bigg, F.R.C.S.

Hy. Frowde and Hodder and Stoughton.

"Abel's Laboratory Handbook of Bacteriology." Translated from the German by M. H. Gordon, M.A., M.D.

T. Nelson and Sons.

"The Hosts of the Lord." By Flora Annie Steel. Nelson's Library.

"The God in the Car." By Anthony Hope. Nelson's Library.

G. P. Putnam's Sons.

"Diagnosis of Organic Nervous Diseases." By Christian A. Herter, M.D. Revised and enlarged by L. Pierce Clark, M.D.

C. Griffin and Co.

"Medical Ethics." By R. Saundby, M.D.

Daly and Co.

"Chats about Wine." By C. E. Hawker. Price 2s. 6d.

Longmans, Green and Co.

"The King's College Hospital Book of Cooking Recipes."

J. and A. Churchill.

"The Borderland of Epilepsy," by Sir Wm. Gowers, M.D.

